# Amifostine Protection Against Mitomycin-induced Chromosomal Breakage in Fanconi Anaemia Lymphocytes

**DOI:** 10.3390/molecules13081759

**Published:** 2008-08-21

**Authors:** Ricardo M. Camelo, Fernanda S. G. Kehdy, Carlos E. Salas, Miriam T. P. Lopes

**Affiliations:** 1 Department of Pharmacology, Instituto de Ciências Biológicas, Universidade Federal de Minas Gerais, Belo Horizonte, CEP 31270-901, Brazil; 2 Departamento Bioquímica e Imunologia, Instituto de Ciências Biológicas, Universidade Federal Minas Gerais, Antônio Carlos 6627, Belo Horizonte, MG 31270-901, Brazil; 3 Department of General Biology, Instituto de Ciências Biológicas, Universidade Federal de Minas Gerais, Belo Horizonte, CEP 31270-901, Brazil

**Keywords:** Fanconi anaemia, amifostine, chromosomal breakage, reactive oxygen species

## Abstract

Fanconi anaemia (FA) is a rare genetic chromosomal instability syndrome caused by impairment of DNA repair and reactive oxygen species (ROS) imbalance. This disease is also related to bone marrow failure and cancer. Treatment of these complications with radiation and alkylating agents may enhance chromosomal breakage. We have evaluated the effect of amifostine (AMF) on basal and mitomycin C (MMC)-induced chromosomal breakage in FA blood cells using the micronucleus assay. The basal micronuclei count was higher among FA patients than healthy subjects. Pre-treatment with AMF significantly inhibited micronucleation induced by MMC in healthy subjects (23.4 ± 4.0 – MMC vs 12.3 ± 2.9 – AMF → MMC) MN/1000CB, p < 0.01, one way ANOVA) as well as in FA patients (80.0 ± 5.8 – MMC vs 40.1 ± 5.8 – AMF → MMC) MN/1000CB, p < 0.01, ANOVA). Release of ROS by peripheral blood mononuclear cells treated with AMF → MMC and measured by chemoluminometry showed that AMF-protection was statistically higher among FA patients than in healthy individuals. Based on these results we suggest that AMF prevents chromosomal breakage induced by MMC, probably by its antioxidant effect.

## Introduction

Fanconi anaemia (FA) is a rare recessive inherited syndrome characterised by chromosomal instability, cellular hypersensitivity to DNA cross linking agents, progressive pancytopenia, congenital malformations and increased predisposition to cancers [1]. The exact pathogenesis has not been elucidated until now, but more than ten altered DNA-repair proteins in the FA/BRCA pathway (the route responsible for maintaining the integrity of the human genome) have been described [2,3].

Androgen therapy can improve the outcome [4], but side effects (e.g. hepatocarcinoma) and tolerance problems arise [5], thus, bone marrow transplantation has become the only well-established treatment for the pancytopenia associated with FA [4]. Ever since low-dose radiotherapy and alkylating treatments were introduced [6], the survival rate has increased significantly and life expectancy reached 20-30 years [7]. However, in these individuals a high risk of developing solid cancers still persists [8].

The imbalance between production of reactive oxygen species (ROS) and their detoxification was suggested to be a causing factor [9], as addition of superoxide dismutase or catalase to FA cultured cells reduced the frequency of chromosomal aberrations. Later, Joenje *et al.* found that chromosomal breakage in FA cells augmented with increasing oxygen tension, thus, it was proposed that the primary defect results from a failure to tolerate oxidative stress [10]. On the other hand, antioxidants should help to counter the deleterious effects induced by oxidative stress. Such is the case for mytomicin C (MMC) associated clastogenicity which depends on oxygen levels [11], as this damage decreases upon addition of low-molecular-weight antioxidants [12], or overexpression of thioredoxin [13].

Amifostine (AMF, WR-2721), a phosphorylated aminothiol, is an antioxidant clinically prescribed to prevent the neutropenia-associated events in patients receiving alkylating agents [14]. In experimental animals, Yuhas and Storer showed that treatment with AMF effectively protects normal tissue from the toxicity of therapeutic radiation, without protecting tumours [15]. Once dephosphorylated by the membrane-bound alkaline phosphatase (ALP), AMF is activated to a free thiol form (WR-1065), which is preferentially up taken by normal cells, since ALP is more active and efficiently expressed in normal rather than neoplastic tissue [16,17]. Nagy *et al.* subsequently showed that AMF provides protection against the mutagenic effects of cisplatin, evaluated by the mutation rate of *HPRT* in V79 Chinese hamster cells [18].

The aim of this study was to evaluate the cytoprotective effect of AMF on spontaneous or MMC-induced chromosomal damage in peripheral lymphocytes from FA patients. To identify a possible mechanism of action for AMF, ROS emission was studied following AMF exposure.

## Results and Discussion

We examined blood from 7 FA patients and 6 healthy volunteers, grouped in 5 for each assay (Table 1). The diagnosis of FA patients was based on clinical criteria and positive chromosomal breakage test. Half of FA donors received oxymetholone for at least one period during their treatment. To verify the ALP activity in PBMC, the NBT-BCIP metabolization method was employed.

**Table 1:** Profile of subjects participating in the study.

Control probands			FA probands					
Code	Age	Sex	Code	Age	Sex	Chromosomal breakage		OXM
						Spontaneous	MMC	
**FMG**	20	M	**ACS**	8	M	0.90	10.32	-
**FSC**	18	F	**CCF**	12	F	0.50	1.16	+
**LES**	15	M	**CBS**	24	F	2.16	12.91	-
**LGBC**	14	M	**EMM**	17	M	1.20	6.12	+
**SSS**	25	F	**RGS**	8	F	0.84	7.00	-
**TMBC**	17	F	**VBM**	7	M	0.80	4.80	-
			**VALA**	13	M	0.92	2.56	+

FA, Fanconi anaemia; M, male; F, female; OXM, oxymetholone + or – indicates prior treatment; MMC, mitomycin C. A spontaneous chromosomal break index ≥ 0.5 or induced break ≥ 1 was required for FA subjects. Normal subjects had chromosomal break index ≤ 0.1 (not shown)

**Figure 1:**
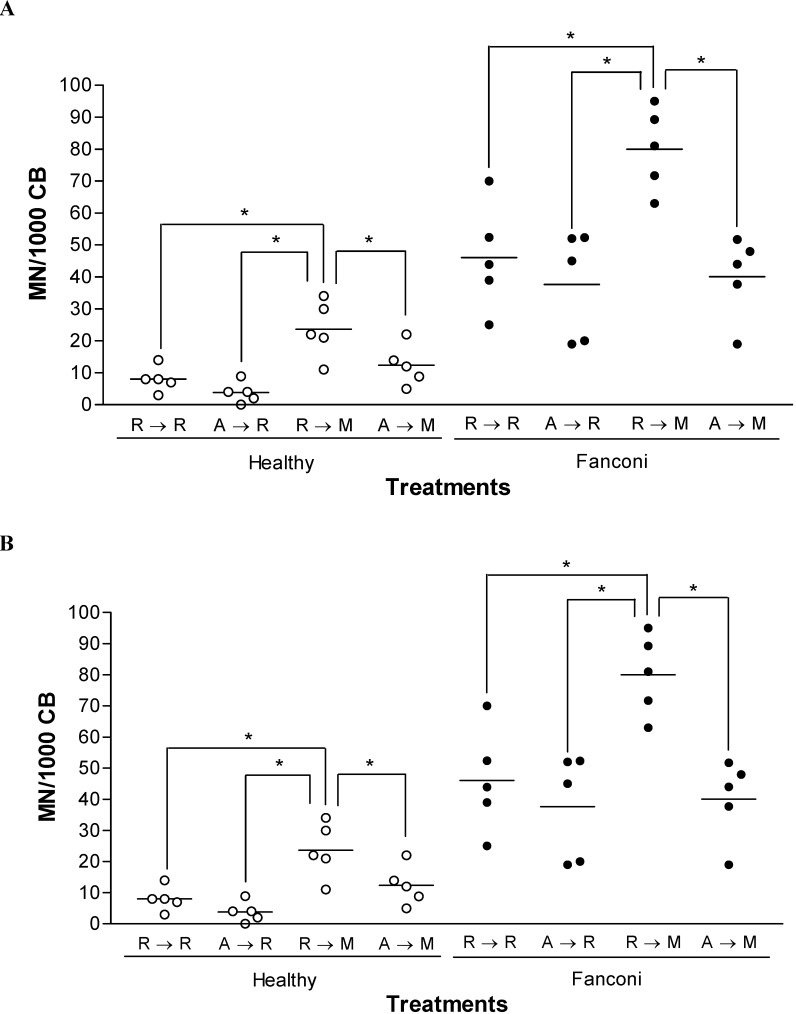
Protective effect of amifostine on mitomycin-induced micronucleation in FA peripheral blood lymphocytes.

ALP activities from healthy and FA PBMC did not show significant differences (1.1 ± 0.2 *vs.* 1.6 ± 0.4, respectively, data not shown). The effect of AMF on spontaneous and MMC-induced chromosomal breakage was studied in lymphocytes from FA or healthy subjects, by the cytokinesis-block micronucleus test. Mean frequencies of MN are shown in Figure 1A. Basal levels of MN were higher among FA subjects than control volunteers (*p* < 0.05, unpaired Student’s *t* test). Isolated AMF treatment did not significantly change the basal frequencies in either group, but, isolated MMC treatment significantly enhanced the MN frequencies from both groups (*p* < 0.05, one way ANOVA). The enhancement in MN frequency induced by MMC was significantly higher among healthy volunteers than FA subjects (Figure 1B, *p* < 0.05, unpaired Student’s *t* test). However, cells pre-treated with AMF for 30 min prevented the increase in MN frequency induced by MMC (Figure 1A), and this protection was similar for both groups (Figure 1B). 

Cells were exposed to 1.2 mg/mL amifostine (A) or RPMI 1640 vehicle (R) for 30 min and then to 150 ng/mL mitomycin (M) or vehicle for 60 min. **R→R**, RPMI 1640 followed by RPMI 1640; **A→R**, amifostine followed by RPMI 1640; **R→M**, RPMI 1640 followed by mitomycin; **A→M**, amifostine followed by mitomycin. Cytochalasin B was added to drug-free medium for 48 h. Binucleated cells (BC) were analysed according to the micronucleus test. Figure 1A represents the absolute frequency of micronucleation and Figure 1B refers to the relative protection by amifostine. One way ANOVA test (Figure 1A) or unpaired Student’s *t* test (Figure 1B), *p* < 0.05 (*), n = 5 individuals per treatment.

**Figure 2:**
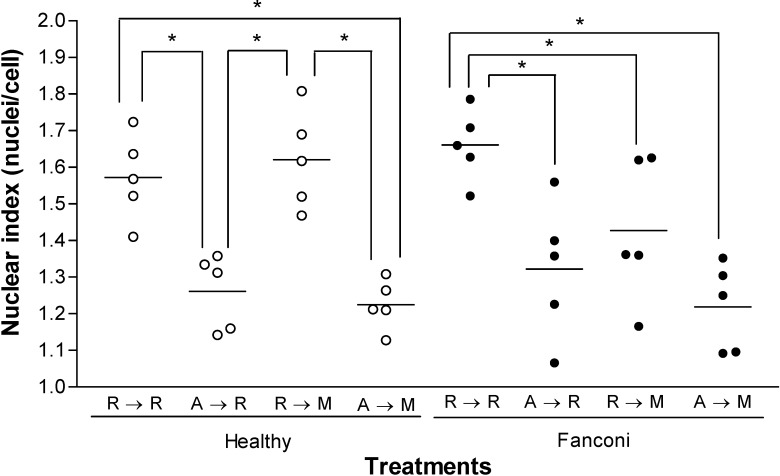
The nuclear index of lymphocytes from healthy and Fanconi anaemia individuals.

Approximately one mitotic event occurred during the experimental period (1.57 ± 0.05 nuclei/cell, for the control group and 1.66 ± 0.04 nuclei/cell in FA probands; Figure 2). Exposure to AMF, followed or not by MMC, prolonged the doubling time in both groups, while MMC alone selectively retarded the cell cycle in affected subjects (*p* < 0.05, one way ANOVA). No differences in cell viability due to apoptosis or necrosis between treatments influenced cell cycle progression (data not shown).

Cells were exposed to 1.2 mg/mL amifostine (A) or RPMI 1640 vehicle (R) for 30 min and then to 150 ng/mL mitomycin (M) or vehicle for 60 min. **R→R**, RPMI 1640 followed by RPMI 1640; **A→R**, amifostine followed by RPMI 1640; **R→M**, RPMI 1640 followed by mitomycin; **A→M**, amifostine followed by mitomycin. Cytochalasin B was added in drug-free medium for 48 h. The number of main nuclei per cell was recorded in 500 viable lymphocytes. One way ANOVA test, *p* < 0.05 (*), n = 5 individuals per treatment.

**Figure 3:**
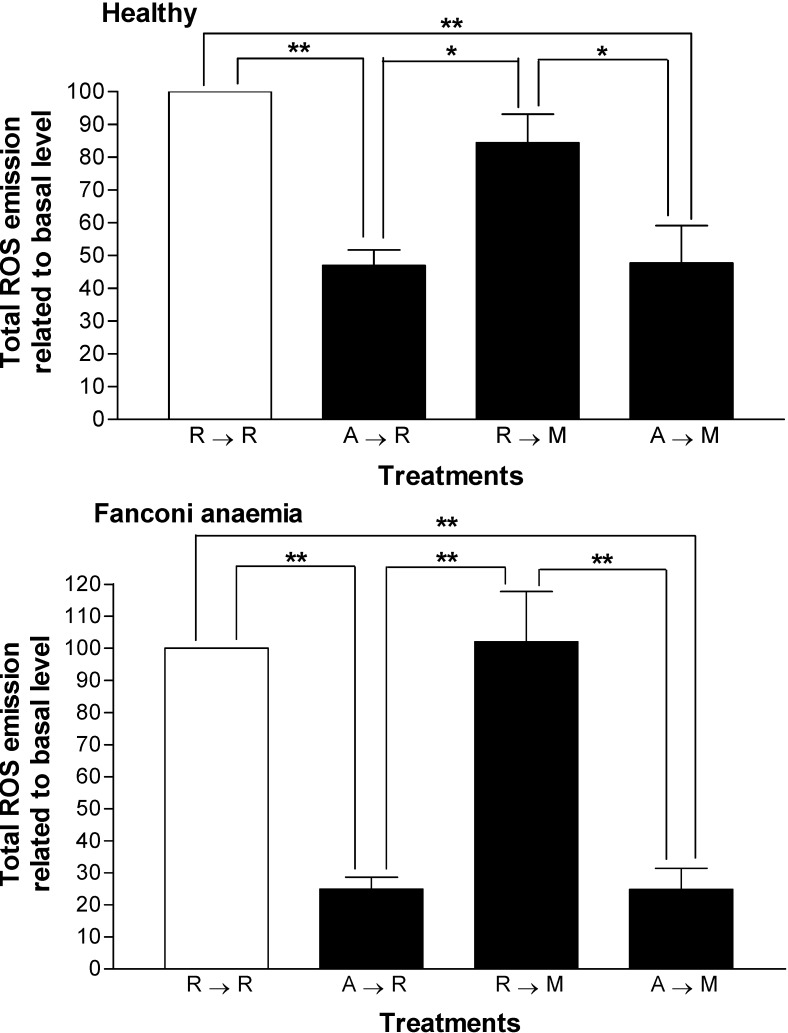
Amifostine effect on the emission of reactive oxygen species (ROS) by peripheral blood mononuclear cells in healthy and Fanconi anemia subjects.

In order to explain the AMF-protection against MMC-induced micronucleation, ROS emission was measured in PBMC from healthy and FA volunteers (Figure 3). AMF significantly reduced ROS emission in both control and FA PBMC (*p* < 0.05, one way ANOVA). Treatment with MMC did not affect the relative energy emission from either group, but, treatment with AMF inhibited ROS release from control and FA PBMC, both, during isolated exposure to AMF or in conjunction with MMC (Figure 3, *p* < 0.05, one way ANOVA). Protection was significantly higher in PBMC from FA volunteers; 76.3 ± 4.9% *vs* 44.4 ± 10.9% for healthy probands, *p* < 0.05, Student’s *t* test).

Cells were exposed to 1.2 mg/mL amifostine (A) or RPMI 1640 vehicle (R) for 30 min and then to 150 ng/mL mitomycin (M) or vehicle for 220 min. **R→R**, RPMI 1640 followed by RPMI 1640; **A→R**, amifostine followed by RPMI 1640; **R→M**, RPMI 1640 followed by mitomycin; **A→M**, amifostine followed by mitomycin. ROS emission was measured by the luminol chemiluminescence method and total emission by PBMC exposed to vehicle only (basal) was considered 100%. One way ANOVA, *p* < 0.01 (*), *p* < 0.001 (**), n = 5 individuals per treatment.

The intrinsic enhanced chromosomal sensitivity to radiation and alkylating agents in FA constitutes an important risk factor for mutagenesis and/or carcinogenesis during the conditioning regimen for bone marrow transplantation and during conventional treatment of cancers afflicting these individuals. Based on this notion, we tested the protective effect of AMF against chromosomal breakage induced by MMC in peripheral blood lymphocytes of FA patients.

The micronucleus test is employed as a tool for measuring mutagenesis in several cell types, including FA cells [13,20,21]. We used this test to evaluate genoprotection, as an alternative cost-effective method, previously used for such a purpose [19]. In agreement with studies in normal lymphocytes [19], we verified a direct relation between spontaneous micronucleation in FA lymphocytes and clastogenicity assessed by the cytogenetic assay used as one of the diagnosis tests for this condition (*p* < 0.05, *r^2^* = 0.94, data not shown).

AMF significantly prevented MMC-induced chromosomal breakage in peripheral blood lymphocytes from both, healthy subjects and FA patients, but did not reduce spontaneous FA micronucleation at the basal level (Figure 1). Other researchers showed that AMF was unable to prevent spontaneous micronucleation of normal lymphocytes [22], but inhibited micronucleation induced by alkylating agents [23,24]. Our observation agrees with data from other groups suggesting that AMF significantly reduces the carcinogenic, mutagenic and clastogenic effects of cancer therapy [22,25,26,27]. Moreover, extensive *in vitro* and *in vivo* evaluations of AMF against radiation- and chemotherapy-induced mutations at the *HGPRT* locus in V79 cells and radiation-induced carcinogenesis confirm the genoprotective effects of the aminothiol [25,26,28].

The basis for selective cytoprotection of normal tissue by AMF is explained by its unique systemic and tissue-distribution pharmacokinetics [29]. A high concentration of ALP, the AMF activating enzyme, occurs in capillaries of normal tissues [16,30], while a lower level and activity of ALP are observed in the fragile endothelium of cancer vessels due to the more acidic environment [17]. Consequently, AMF acts preferentially against chemotherapy-induced DNA damage in normal tissues, rather than in cancer cells [31]. These data explain why AMF pretreatment of tumour-bearing animals increases the therapeutic index of anticancer drugs [32]. Our results demonstrate that PBMC from both groups of subjects exhibit similar ALP activity, thus yielding similar AMF concentrations on each group, hence, the protective effect of AMF must be consequence of a different mechanism.

An important mechanism of cytoprotection involves the ability of free thiol groups to act as scavengers for oxygen free radicals, such as those derived from specific drugs or radiation therapy [33]. AMF significantly prevented the release of reactive oxygen species in both control and FA individuals (Figure 2). The ROS emission induced in both groups by AMF was unchanged by the addition of MMC. However, the antioxidative effect by AMF was stronger in FA cells compared to its control (Figure 3, A→M). This evidence signals an oxidative imbalance in FA cells exposed to oxidative stress. In line with these data, prior evidence showed that low levels of thioredoxin in FA fibroblasts and consequent lost of sensitivity to MMC, was restored by thioredoxin overexpression [34]. The vulnerability to oxidative stress by FA is explained, as some FANC proteins are linked to redox pathways: i.e., FANCA, FANCC, and FANCG [35,36,37,38]. The absence of ROS changes in R→M compared to R→R in normal and Fanconi probands (Figure 3) is justified by the low MMC dose (0.5 μM) used, as other authors reported that ROS formation at low MMC dose is not significant [11]. In this case the clastogenic effect is associated with formation of ●MMC radical, a species that also reacts with DNA.

Although the basal nuclear indices were similar in both experimental groups (Figure 2 RPMI → RPMI), the longer arrest on the duplication time in FA cells containing MMC could result from impairment in DNA repair, as suggested before [39]. Meanwhile, North *et al.* suggested a direct genotoxic effect of AMF which could interrupt cell cycle progression, but we could not confirm this effect by the micronucleus test [40]. The delaying effect by AMF is unrelated to cell apoptosis or necrosis, since control assays (morphological Giemsa staining; data not shown) failed to detect significant changes of these phenomena. DNA binding to AMF or its metabolites to protect from ROS attack may interfere in the activity of some enzymes, such as topoisomerases and the interference of some transcription factors, such as NF-κB, AP-1 and p53 could explain the arrest observed in the present study [41,42,43]. Interestingly, a recent report shows that AMF improves conditioning of FA bone marrow cells before transplantation [44].

The American Society of Clinical Oncology approved the use of AMF to reduce the neutropenia-associated events in patients receiving alkylating agents, during chemotherapeutic treatment [14]. In addition, AMF and its disulfide WR-33278 display myelostimulatory properties on normal stem cells, likely by its structural similarity with endogenous polyamines, usually related to cell proliferation and differentiation [45]. 

Since Nordenson described the correction of chromosomal abnormalities in FA cells exposed to superoxide dismutase and catalase *in vitro*, many studies reported the genoprotective effect by enzyme supplementation or antioxidants [9,12,13]. However, a single report describes a reduction of chromosomal instability in a FA patient treated with rutin, leading to suggestion of a diet rich in antioxidants for these individuals [46,47].

The protective effects of AMF and/or its metabolites on DNA alkylation or free radical induced DNA damage not only have implications on acute cytotoxic manifestations from therapy, but also against potential genotoxic effects of oncostatic therapies resulting in secondary malignancies [25]. To our knowledge, this is the first study involving AMF as a cytoprotector for FA. We have shown that AMF can prevent MMC-induced chromosomal breakage, probably by reducing the pro-oxidative state of mononuclear cells. Future research should investigate the levels of antioxidants in FA cells and the modulation induced by AMF exposition. Clinical trials with AMF should be encouraged before bone marrow conditioning or prior to chemo- or radiotherapies used for cancer treatment.

## Experimental

### Subject eligibility

A total of seven FA patients (Table 1, five randomly chosen for each assay) were selected according to the following diagnostic criteria: (a) clinical manifestations of the disease (haematological abnormalities and/or congenital anomalies typical of FA) and (b) cytogenetic studies (spontaneous chromatid breakage and hypersensitivity to MMC) in phytohaemagglutinin-stimulated lymphocytes. Six normal, healthy- and sex-matched volunteers (Table 1, five randomly chosen for each assay) were recruited as controls. Their spontaneous chromosomal break index was lower than 0.1. General exclusion criteria were: (1) inpatients, (2) prior bone marrow transplantation, (3) previous or current diagnosis of malignancy and (4) tabagism or habitual alcoholism. A sample from every subject was used in each experiment. The study received approval from the Committee on Ethics and Research of the Federal University of Minas Gerais, Belo Horizonte, Brazil. Free informed consent was obtained from all subjects.

### Sample collection and isolation of peripheral blood mononuclear cells (PBMC)

Peripheral blood samples (3-4 mL in EDTA-vacutainer tubes) were obtained when patients attended a routine physician visit. PBMC from FA patients and control subjects were isolated by Hystopaque^®^ (Sigma) sedimentation and washed twice in phenol red-free RPMI 1640.

### Chemicals and culture medium

Cytochalasin B (Cyt-B; Sigma); phytohaemagglutinin A (PHA; GIBCO); luminol (5-amine-2,3-dihydro-1,4-phtalazinedione, Sigma); nitrobluetetrazolium chloride (NBT; Gibco); *p*-toluidine 5-bromo-4-chloro-3-indolylphosphate (BCIP; Gibco); amifostine (AMF, Ethyol^®^; Schering-Plough); mitomycin C (MMC, Mytocin^®^; Bristol-Myers) were used in this study.

Lymphocytes were cultivated in RPMI 1640 medium, supplemented with 7.5% NaHCO_3_ (w/v), 20% foetal bovine serum (v/v) (FBS; Gibco), 2 mmol/L l-glutamine (Gln; Gibco) and antibiotics (100 U/mL penicillin, 100 μg/mL streptomycin and 25 μg/mL amphotericine B; Gibco).

### Alkaline phosphatase activity

Erythrocytes were removed from PBMC pellet by rinsing with haemolysis buffer (8.29 g/L NH_4_Cl, 1.0 g/L KHCO_3_, 37.2 mg/L EDTA, in deionized water, pH 7.2-7.4). The pellet was then resuspended in ALP buffer (12.1 g/L Tris base – TRIZMA®BASE, Sigma –, 5.85 g/L NaCl, 4.75 g/L MgCl_2_.6H_2_O, in deionized water, pH 9.0) at a final density of 5 x 10^6^ cells/mL.

NBT (330 ng/mL) and then BCIP (65 ng/mL) were added and, after 3 h in the darkness (37^o^C), 3-5 aliquots (100-200 μL) were distributed on a 24-well plate and then 10% SDS-HCl (v/v) was added and incubated for 18 h at 37^o^C. The colorimetric reaction was spectrophotometrically measured at λ = 570 nm) and blanks containing buffer plus NBT and BCIP were subtracted from experimental points.

### Micronucleus assay

The micronucleus (MN) assay was performed according to Fenech *et al.* (19) with modifications. Cells were distributed into 8 tubes (10^6^ cells/mL for control probands or the whole PBMC suspension for FA patients) and incubated at 37^o^C and 5% (v/v) CO_2_-humidified atmosphere, in RPMI 1640 medium (Gibco) containing 20% (v/v) FBS (Gibco) and antibiotics (Gibco), 2 mmol/L Gln (Gibco) and 4% (v/v) PHA (Gibco). After 44 h incubation, medium was changed and cells were exposed to 1.2 mg/mL AMF (the highest concentration without toxic effect, according to guidelines from Organization for Economic Co-Operation and Development, 1995, or free medium for 30 min followed by 150 mg/mL MMC, or free medium for additional 60 min. Cells were washed twice with free medium and Cyt-B (6 μg/mL) was added for 48 h. Each assay was done in duplicate and pooled before fixation.

Cells were harvested by centrifugation and fixed in 3:1 70% methanol: acetic acid (v/v) and distributed onto two slides. Cells were stained with 4% (v/v) Giemsa (Gibco) for 15 min and nuclear changes microscopically identified (1000*x* by immersion) by a trained person who did not know to which proband the slide belongs to.

From 1-4 nuclei were scored in 500 viable lymphocytes and the nuclear index (NI) calculated according to equation (1):

NI = (MC + 2 x BC + 3 x TC + 4 x QC) / total viable cells
(1)

where MC, BC, TC and QC represents mono-, bi-, tri- or tetranucleated cells, respectively.

Scoring of MN was performed in BC. A total of 1000 BC with distinct, homogeneously coloured and sized nuclei were scored on each proband, according to the following criteria: (1) rounded body(ies) with a diameter between 1/16 and 1/3 of the main nuclei; (2) non-refractivity; (3) staining not darker than the main nuclei; (4) the main nuclei can be contacting but not overlapping.

### Chemiluminescence analysis

PBMC were washed twice in phenol red-free RPMI 1640 and adjusted to a final concentration of up to 5 x 10^6^ cells/mL. Each condition was assayed in duplicate in a 96-well tarnished plate to which luminol was added to a final concentration of 0.01 mol/L at time t = 0 min. At this time, cells were exposed for 30 min to 1.2 mg/mL AMF or medium (vehicle) and then to 150 ng/mL MMC or vehicle for the last 220 min. ROS emission was measured by a chemiluminometer (LumiCount Microplate Luminometer, Packard) in the darkness at 37^o^C, shaking the plate for 1 s before each reading and with the following instrument settings: gain of 32, 1100 V at the photomultiplier tube and 0.5 s for photon capture. Reading was made with 2-min intervals during the first 30 min followed by 45 readings at 5 min intervals. The emission produced by a blank containing medium plus luminol was subtracted from experimental values. The total emission (luminosity) was calculated as the area under the curve and plotted as ROS emission *vs.* time. The basal emission obtained by treatment with RPMI 1640 → RPMI 1640) was assigned 100% and used as reference.

### Statistical analysis

Differences between the experimental values, means and variances were statistically analysed by PRISM (GraphPad, San Diego, CA) software. Statistical analysis was performed by one way ANOVA followed by unpaired Student’s *t* test or by Newman-Keuls multiple comparison test. A *p* value ≤ 0.05 was considered statistically significant.
